# Conditional deletion of *Ccl2* in smooth muscle cells does not reduce early atherosclerosis in mice

**DOI:** 10.1016/j.athplu.2023.12.004

**Published:** 2023-12-20

**Authors:** Stine Gunnersen, Jeong Tangkjær Shim, Fan Liu, Uwe J.F. Tietge, Charlotte Brandt Sørensen, Jacob Fog Bentzon

**Affiliations:** a Atherosclerosis Research Unit, Department of Clinical Medicine, Aarhus University, Palle Juul-Jensens Boulevard 11, 8200 Aarhus N, Denmark; b Department of Cardiology, Aarhus University Hospital, Palle Juul-Jensens Boulevard 99, 8200 Aarhus N, Denmark; c Division of Clinical Chemistry, Department of Laboratory Medicine, Karolinska Institutet, 14183 Stockholm, Sweden; d Department of Pediatrics, University of Groningen, University Medical Center Groningen, 9700 RB Groningen, The Netherlands; e Clinical Chemistry, Karolinska University Laboratory, Karolinska University Hospital, SE-14186 Stockholm, Sweden; f Centro Nacional de Investigaciones Cardiovasculares Carlos III (CNIC), Calle de Melchor Fernández Almagro, 3, 28029 Madrid, Spain; g Steno Diabetes Center Aarhus, Aarhus University Hospital, Palle Juul-Jensens Boulevard 11, 8200 Aarhus N, Denmark

**Keywords:** Smooth muscle cells, CCL2, Inducible knockout, Inflammation, Atherosclerosis

## Abstract

**Background and aims:**

C–C motif chemokine ligand 2 (CCL2) is a pro-inflammatory chemokine important for monocyte recruitment to the arterial wall and atherosclerotic plaques. Global knockout of *Ccl2* reduces plaque formation and macrophage content in mice, but the importance of different plaque cell types in mediating this effect has not been resolved. Smooth muscle cells (SMCs) can adopt a potentially pro-inflammatory function with expression of CCL2. The present study aimed to test the hypothesis that SMC-secreted CCL2 is involved in early atherogenesis in mice.

**Methods:**

SMC-restricted Cre recombinase was activated at 6 weeks of age in mice with homozygous floxed or wildtype *Ccl2* alleles. Separate experiments in mice lacking the Cre recombinase transgene were conducted to control for genetic background effects. Hypercholesterolemia and atherosclerosis were induced by a tail vein injection of recombinant adeno-associated virus (rAAV) encoding proprotein convertase subtilisin/kexin type 9 (PCSK9) and a high-fat diet for 12 weeks.

**Results:**

Unexpectedly, mice with SMC-specific *Ccl2* deletion developed higher levels of plasma cholesterol and larger atherosclerotic plaques with more macrophages compared with wild-type littermates. When total cholesterol levels were incorporated into the statistical analysis, none of the effects on plaque development between groups remained significant. Importantly, changes in plasma cholesterol and atherosclerosis remained in mice lacking Cre recombinase indicating that they were not caused by SMC-specific CCL2 deletion but by effects of the floxed allele or passenger genes.

**Conclusions:**

SMC-specific deficiency of *Ccl2* does not significantly affect early plaque development in hypercholesterolemic mice.

## Highlights

• SMCs express CCL2 in human plaques and upon inflammatory activation of a murine SMC line in vitro.
• *Ccl2* deletion in SMCs does not influence the size or composition of atherosclerotic plaques in mice.
• Control studies showed that an initially observed difference in atherosclerosis was a genetic background effect.

## 1 Introduction

Atherosclerosis is a low-grade, non-resolving inflammatory disease driven by retention of low-density lipoproteins (LDL) in the arterial wall [1]. Inhibition of inflammatory pathways reduces plaque progression in experimental models and protects against the incidence of clinical atherosclerotic events in patients with prior myocardial infarction [2,3]. Blocking inflammation broadly in the body, however, adversely affects defenses against infection. Research is needed to identify mechanisms that locally control inflammatory cells to devise potent anti-inflammatory therapies that are specific or selective for atherosclerosis [1,4].

C–C motif chemokine ligand 2 (CCL2) is a pro-inflammatory chemokine secreted by multiple cell types, which is central in the recruitment of monocytes to tissues and has a clear causal role in atherosclerosis [4]. Global deficiency of *Ccl2* in *Apoe*^−/−^ mice, *Ldlr*^−/−^ mice, and mice overexpressing apoB consistently inhibited atherogenesis and reduced plaque content of macrophages [[5], [6], [7]]. Blocking *Ccl2* activity in mice with advanced atherosclerosis reduced further plaque progression and changed plaque composition to contain fewer macrophages, more SMCs, and more collagen [8]. These studies indicate a continued role of CCL2 that extends beyond disease initiation. CCL2 is also expressed in human atherosclerotic plaques and associated with features of plaque vulnerability [9], and *CCL2* polymorphisms and elevated CCL2 plasma levels are associated with an increased risk of cardiovascular disease and mortality [[9], [10], [11]].

Expression in myeloid cells accounts for a part of the atherosclerosis-promoting effect of CCL2 in mice [12]. However, single-cell gene expression profiling of atherosclerotic arteries and other studies has revealed widespread expression of CCL2 in SMC lineage cells, suggesting that SMC-derived CCL2 may carry some of the proatherogenic effect [[13], [14], [15]]. Two recent reports, published while we were finalizing the present study, investigated this in *Apoe*^*−/−*^ mice but reached different conclusions. One report found a pro-atherogenic role of locally secreted CCL2 from plaque SMC-derived cells, which in some contexts was offset by an inhibitory effect of bone marrow SMC/pericyte-derived CCL2 on monocytosis [16]. The other found exacerbated plaque inflammation and reduced cap SMCs after SMC-specific deletion of CCL2, and suggested that SMC-derived CCL2 is crucial for maintaining niches of homeostatic macrophages in the plaque that dampen chronic inflammation [14].

In the present study, we assessed the importance of SMC-secreted CCL2 for early atherosclerosis in murine atherosclerosis induced by PCSK9 overexpression. We find no effect of SMC-derived CCL2 on plaque development in this model when accounting for background effects of the engineered *Ccl2* allele or its passenger gene variants.

## 2 Materials and methods

Please see an expanded methods section in the Supplemental Material.

### 2.1 Mice

Male *Myh11-*CreER^T2^ mice (B6·FVB-Tg(Myh11-cre/ERT2)1Soff/J, stock no. 019079) expressing tamoxifen-inducible Cre recombinase (*CRE-ERT2)* under the SMC-specific myosin heavy chain 11 (*Myh11*) promotor [17], and *Ccl2*^flx/flx^ mice (B6N·129S1(FVB)-Ccl2tm1.2Tyos/J, stock no. 023347) with *loxP* sites flanking exon 1–2 of the *Ccl2* gene [18] were acquired from Jackson and intercrossed. Experimental mice of our study were male offspring of breeders heterozygous for the floxed *Ccl2* allele. Only males were included because the Myh11-CreER^T2^ transgene is inserted on the Y chromosome. One of the male breeders was found without the Y-linked *Myh11*-*CreER*^*T2*^, presumably due to the exchange in genetic material between pseudoautosomal regions of X and Y chromosomes [19,20], and was used to breed CreER^T2^-negative male mice for control experiments. Animals were housed in groups with unlimited access to tap water and a standard laboratory diet in an enriched environment with standard bedding and nesting material under a 12/12 h day-night cycle in a temperature (20-25 °C) controlled facility.

At 6–8 weeks of age, male *Myh11-*CreER^T2^ x *Ccl2*^flx/flx^ and *Myh11-*CreER^T2^ x *Ccl2*^wt/wt^ littermates were injected i.p. with 1 mg tamoxifen (T5648, Sigma-Aldrich) dissolved in corn oil (P2144, Sigma-Aldrich) daily for 10 days to obtain mice with or without SMC-specific *Ccl2* deletion (*Ccl2*^SMC−KO^ and *Ccl2*^SMC−WT^). Male *Ccl2*^flx/flx^ and *Ccl2*^wt/wt^ littermates were treated similarly. At 12 weeks of age, hypercholesterolemia was induced by a single tail vein injection of rAAV8-D377Y-mPCSK9 virus particles (rAAV-PCSK9, 1 × 10^11^ vector genomes, produced at Vector Core at the University of North Carolina, Chapel Hill, North Carolina, USA) and shifted to a high-fat diet (D12079B, Research Diets Inc.) containing 21 % fat and 0.2 % cholesterol [21]. After 12 weeks of feeding atherosclerosis-promoting diet, the mice were anesthetized with 5 mg pentobarbital and 4 mg lidocain i.p. and terminated by exsanguination. The arterial tree was perfused with Cardioplex for 30 s and 4 % phosphate-buffered formaldehyde (PFA) for 5 min at 100 mmHg through the left ventricle, followed by immersion-fixation in 4 % PFA for 24 h. Animal procedures were approved by The Danish Animal Experiments Inspectorate (license 2015-15-0201-00542) and conducted at Aarhus University following ARRIVE guidelines.

### 2.2 Atherosclerosis analysis

The top half of the heart containing the aortic root was removed and embedded in paraffin. Transversal sections (3 μm) of the aortic root were cut from the distal part of the heart and serial sections were collected at 0 μm, 80 μm, 160 μm and 240 μm from the level of the aortic valve commissures. For quantification of atherosclerosis, sections from all levels (main experiment) or the first two levels (control experiment with Cre-negative mice) were stained by orcein. Sections from the first level (main experiment) were stained for smooth muscle alpha-2 actin (ACTA2) and galectin 3 (LGALS3) using mouse monoclonal anti-ACTA2 (Dako, cat. no. M0851, 1:100) after blocking with Fab fragment (Jackson ImmunoResearch, cat. no. 715-007-003, 1:10) and rat monoclonal IgG2a anti-LGALS3 (Cederlane, cat. no. CL8942AP, 1:250) followed by Alexa 488-conjugated donkey anti-mouse (Invitrogen, cat. no. A21202, 1:400) or Alexa 488-conjugated donkey anti-rat (Invitrogen, cat. no. A21208, 1:400) secondary antibodies. Analysis of stained sections was performed in Fiji (also known as ImageJ) [22].

Aortae were cleaned of all periadventitial fat, cut open longitudinally, stained with Oil Red O for 10 min at 37 °C, washed in isopropanol and phosphate buffered saline (PBS), and mounted on a microscope slide with Aquatex for en face quantification of lesion coverage in the aortic arch down to the supreme intercostal artery. Analysis of the scanned slides was performed in Fiji.

### 2.3 Human atherosclerotic plaques

Human atherosclerotic plaques from the left anterior descending artery obtained from a previously published material [23] were used for immunohistochemical analysis of CCL2 localization using rabbit polyclonal anti-CCL2 (Lifespan Bioscience, cat. no. LS-B10540, 1:50) and Envision + System-HRP (DAB) (Dako, cat. no. K4010). The original material was collected after approval by the regional ethical committee [23]. It was subsequently anonymized and did not require further ethical approval for the use in the present study.

### 2.4 Cell studies

An immortalized murine smooth muscle cell line (MOVAS, ATCC CRL-2797) was maintained in culture in DMEM (Fischer Scientific, cat. no. 41965062) supplemented with 10 % fetal calf serum (Sigma-Aldrich, cat. no. F7524), 1 % penicillin-streptomycin (Invitrogen, cat. no. 15140122) and 1 % gentamycin (G418, Invivogen cat. no. ant-gn-1). Primary human aortic SMCs (hVSMCs), obtained as previously described [24], were kindly provided by Thomas Ledet, Aarhus University. They were maintained in DMEM supplemented with 10 % FCS and 1 % penicillin-streptomycin. Incubation was performed at 37 °C in a humidified atmosphere of 95 % air and 5 % CO_2_ and the medium was changed every 2–3 days. MOVAS cells were seeded for experiments in 12-well plates at a density of 6.25 × 10^3^ cells/cm^2^, and human SMCs were seeded in 6-well plates with a density of 1.6 × 10^4^ cells/cm^2^. The experiments were performed 24 h after seeding the cells. To induce an inflammatory response in hVSMCs and MOVAS cells, the cells were incubated with interleukin-1β (IL-1β, R&D Diagnostics, cat. no. 401-ML-005/CF, 0.01–100 ng/ul) for 24 h. The conditioned media (CM) were then collected and cleared from debris by centrifugation (3,000×*g* for 5 min). CCL2 levels were measured in conditioned media by ELISA (Invitrogen, cat nos. 88-7391-88 and 88-7399-88).

### 2.5 Statistics

All statistical analysis was performed using GraphPad Prism 9 (GraphPad Software Inc.). Data was checked for normal and log-nomal distribution and equal variance and analyzed using the tests indicated in figure legends. Multiple regression analysis was used to analyze associations with plaque data using genotype and plasma cholesterol as independent variables and associations with plasma cholesterol levels (using genotype and plasma PCSK9 concentrations as independent variables). *P* values below 0.05 were defined as statistically significant. Data are shown as mean ± standard error of the mean (SEM) as indicated in each figure legend. The number of animals/samples analyzed is stated in figure legends. In some analyses, data points were not obtained for all mice because of technical failures. All procedures and analyses were performed blinded for the mouse genotype.

## 3 Results

### 3.1 CCL2 is expressed in human atherosclerotic lesions

**Fig. 1:**
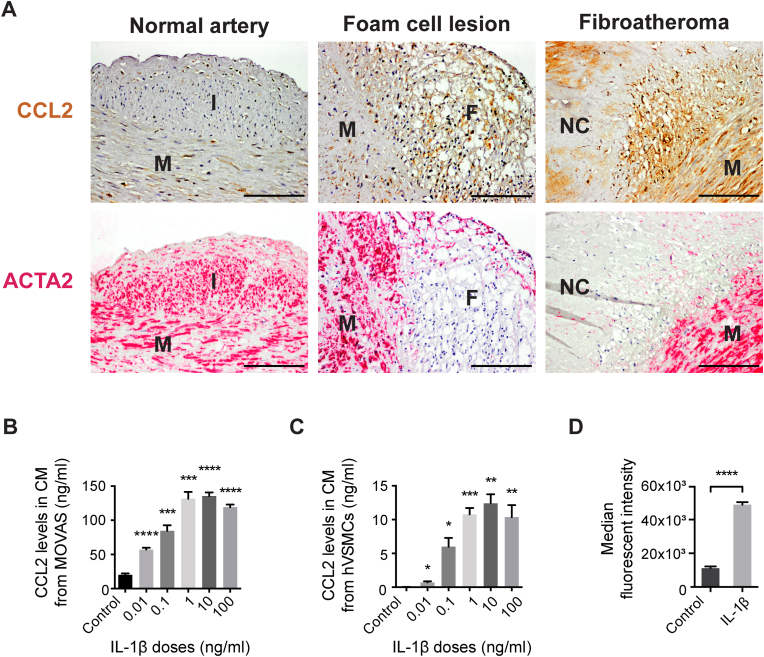
**CCL2 expression in SMC-rich regions of human plaques and CCL2 secretion in SMC cultures.** (A) CCL2 (brown color) and ACTA2 (red color) detected by immunohistochemistry in adjacent sections of normal human artery wall and atherosclerotic lesions of different severity. CCL2 is expressed in media and intima of the normal arterial wall. CCL2 is also expressed in foam cells and underlying medial SMCs, and in active inflammatory areas adjacent to the necrotic core in the fibroatheroma. F, foam cells. I, Intima. M, Media, NC, necrotic core. Scale bars = 100 μm. CCL2 is secreted from cultured SMCs stimulated with IL-1β. CCL2 levels measured in conditioned media (CM) from (B) MOVAS cells (n = 6 replicates per group) and (C) hVSMCs (n = 5–6 replicates per group) treated with IL-1β for 24 h. (D) Flow cytometry measurement of intracellular CCL2 accumulation in MOVAS cells after blocking the secretory pathway by Brefeldin-A. IL-1β stimulation (1 ng/ml) increases CCL2 accumulation measured as median fluorescent intensity (n = 3 replicates per group). Graphs show mean ± SEM. *p < 0.05, **p < 0.01, ***p < 0.001, ****p < 0.0001 by Dunnett's T3 multiple comparisons test following Brown-Forsythe ANOVA (B,C; individual IL-1β dosage groups compared with control) and unpaired Student's *t*-test (D). (For interpretation of the references to color in this figure legend, the reader is referred to the Web version of this article.)

To evaluate the expression pattern of CCL2 during human atherogenesis, we performed CCL2 immunohistochemistry in human coronary lesions of different severity (Fig. 1A). CCL2 was found expressed in normal human coronary artery in both the medial and intimal layer. In early lesions, CCL2 was present in foam cells, but also in the underlying artery wall. In advanced atherosclerotic lesions, CCL2 was detected in areas adjacent to the necrotic core, while CCL2 expression in the medial layer was diminished. Staining of adjacent sections with ACTA2 showed considerable overlap with the pattern of CCL2 staining suggesting widespread SMC expression of CCL2 in normal media/intima as well as in developing plaques.

### 3.2 Human and murine SMCs in culture can be stimulated to express CCL2

Previous studies have reported CCL2 secretion from murine SMCs upon stimulation [24]. To confirm this, we stimulated human VSMCs and a murine SMC line (MOVAS) with IL-1β and found that the secretion of CCL2 in the conditioned media of both increased dose-dependently (Fig. 1B and C). Also, assessed by flow cytometry, we found an increase in protein expression of CCL2 in response to IL-1β stimulation of MOVAS cells (Fig. 1D).

### 3.3 SMC-specific conditional knockout of *Ccl2* in hypercholesterolemic mice

**Fig. 2:**
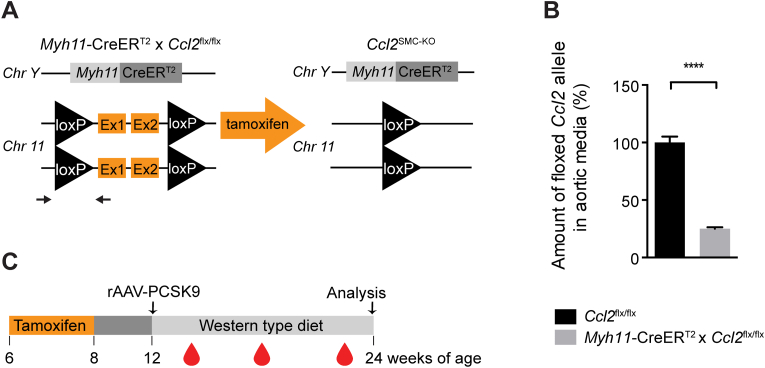
**Experimental design.** (A) Illustration of the genetic alterations in *Myh11-*CreER^T2^ x *Ccl2*^flx/flx^ mice that lead to SMC-specific knockout of *Ccl2* after tamoxifen injection. Black arrows flanking the left loxP site indicate primers used for detecting recombination by qPCR. (B) Recombination efficiency was measured as the reduction in (non-recombined) floxed CCL2 alleles in genomic DNA of the aortic media in *Myh11-*CreER^T2^ x *Ccl2*^flx/flx^ (n = 21, grey bar) compared with *Ccl2*^flx/flx^ (n = 15, black bar) mice. The graph shows mean ± SEM. ****p < 0.0001 by unpaired Welch *t*-test. (C) Schematic illustration of the mouse experiment showing the timing of tamoxifen injections (10 daily doses between 6 and 8 weeks of age), induction of LDL receptor-deficiency by a single tail-vein injection of rAAV8-D377Y-mPCSK9 at 12 weeks of age, and final analysis after feeding Western-type diet for 12 weeks. Blood samples were collected 2, 6, and 11 weeks after rAAV-PCSK9 injection.

Modulation to a CCL2-secreting SMC phenotype by local IL-1β or other pro-inflammatory stimuli could facilitate ongoing inflammation in the developing atherosclerotic lesion. To test this hypothesis *in vivo*, we crossed mice with SMC-restricted CreER^T2^ expression and either homozygous floxed or wildtype *Ccl2* alleles to obtain mice with SMC-specific deletion of *Ccl2* (*Ccl2*^SMC−KO^) and littermate controls (*Ccl2*^SMC−WT^) (Fig. 2A). Mean allelic recombination efficiency was 75.9 ± 1.3 % (mean ± SEM) (Fig. 2B). Because recombination was determined in aortic samples after mechanical removal of the adventitia and endothelium, the analyzed cells may have contained a small amount of non-SMC cell types causing some underestimation of recombination efficiency.

### 3.4 Increased plasma cholesterol levels in *Ccl2*^SMC−KO^ mice

**Fig. 3:**
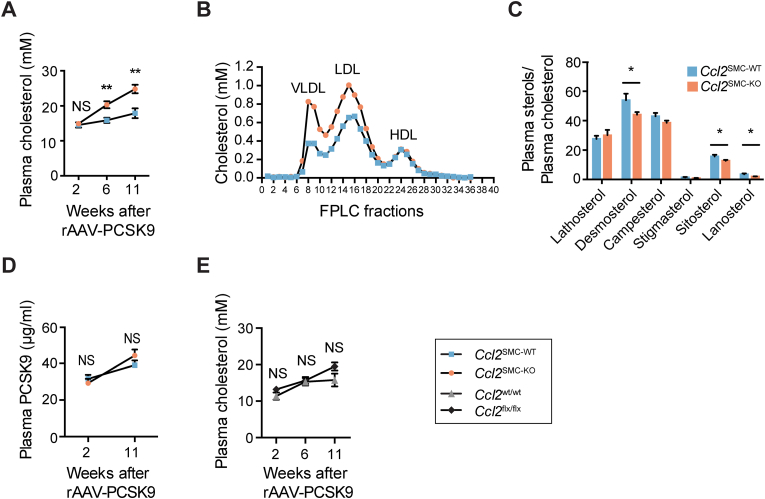
**Plasma cholesterol phenotype.** (A) Plasma cholesterol levels were initially similar but significantly increased in *Ccl2*^SMC−KO^ (n = 22, orange circles) compared with *Ccl2*^SMC−WT^ (n = 24, blue squares) mice at 6 and 11 weeks after rAAV-PCSK9 injection. (B) Analysis of size-fractionated lipoproteins by FPLC at 12 weeks after rAAV-PCSK9 injection revealed increases in cholesterol in VLDL- and LDL-sized, but not HDL-sized, lipoproteins. (C) Sterol analysis for markers of cholesterol biosynthesis (lathosterol and desmosterol) or cholesterol absorption (campesterol, stigmasterol, sitosterol, lanosterol) showed no significant changes or changes opposite the direction that could explain the increased plasma cholesterol levels in *Ccl2*^SMC−KO^ (n = 23) compared with *Ccl2*^SMC−WT^ mice (n = 21). Plasma PCSK9 levels were not different between *Ccl2*^SMC−WT^ (n = 22) and *Ccl2*^SMC−KO^ mice (n = 24) (D). (E) Plasma cholesterol levels were numerical increased, though not statistically significant, in *Cre*-negative *Ccl2*^flx/flx^ (n = 15, black squares) compared with *Ccl2*^wt/wt^ (n = 17, grey triangles) mice 11 weeks after rAAV-PCSK9 injection. Graphs show mean ± SEM. *p < 0.05; **p < 0.01; NS, not statistically significant in a mixed-effects model for repeated measurements with Šidák's multiple comparison test (A,D,E) and Welch *t*-test (C). (For interpretation of the references to color in this figure legend, the reader is referred to the Web version of this article.)

Atherosclerosis was induced in *Ccl2*^SMC−KO^ and *Ccl2*^SMC−WT^ mice by rAAV-PCSK9 injection followed by feeding a high-fat diet for 12 weeks (Fig. 2C). Plasma cholesterol was similar in the two genotypes after 2 weeks but significantly higher in *Ccl2*^SMC−KO^ mice than *Ccl2*^SMC−WT^ mice at both 6 and 11 weeks (Fig. 3A). Analysis of size-fractioned lipoproteins measured at 12 weeks showed that the increased cholesterol in *Ccl2*^SMC−KO^ mice was in apoB-containing lipoproteins (VLDL and LDL) (Fig. 3B).

Sterol analysis was performed to provide clues to the source of the increased plasma cholesterol in *Ccl2*^SMC−KO^ mice (Fig. 3C) [25]. Markers for the rate of cholesterol synthesis (lathosterol and desmosterol) and intestinal cholesterol absorption (campesterol, stigmasterol, sitosterol, lanosterol) were either unchanged or reduced in plasma of *Ccl2*^SMC−KO^ compared with *Ccl2*^SMC−WT^ mice. These observations instead pointed towards a potential difference in LDL clearance, which could be caused by a difference in rAAV8 transduction efficiencies leading to differences in PCSK9 levels. We therefore measured plasma PCSK9 levels in samples taken at 2 and 11 weeks after rAAV8. Plasma PCSK9 levels increased from 2 to 11 weeks, but no statistically significant differences were found between *Ccl2*^SMC−KO^ and *Ccl2*^SMC−WT^ mice (Fig. 3D). Furthermore, introducing plasma PCSK9 levels in multiple regression analysis did not weaken the link between genotype and plasma cholesterol levels (Supplemental Table 1).

Although the floxed *Ccl2* mice were already backcrossed into the B6 mouse background 10 times when we acquired them and further 3–4 times in our facility, a region around the *Ccl2* locus will be derived from the 129/SVJ embryonic stem cells in which the *Ccl2* gene targeting was performed [26]. This passenger gene region could potentially harbor plasma cholesterol regulating loci. Furthermore, the genetic modification of the *Ccl2* locus could affect neighboring gene expression in any cell type, which in theory could impact lipoprotein metabolism. To explore this, we also analyzed plasma lipids in CreER^T2^-negative *Ccl2*^flx/flx^ and *Ccl2*^wt/wt^ mice that had been subjected to a similar regimen of rAAV8-PCSK9 treatment and high-fat diet. We found a numerical increase in plasma cholesterol levels in *Ccl2*^flx/flx^ compared with *Ccl2*^wt/wt^ at the last time point (Fig. 3E). Although this difference was not significant (*p* = 0.08*)*, it is consistent with genetic background effects, rather than the SMC-specific *Ccl2* deletion, being the underlying cause of the differences in plasma cholesterol.

### 3.5 Atherosclerosis development

**Fig. 4:**
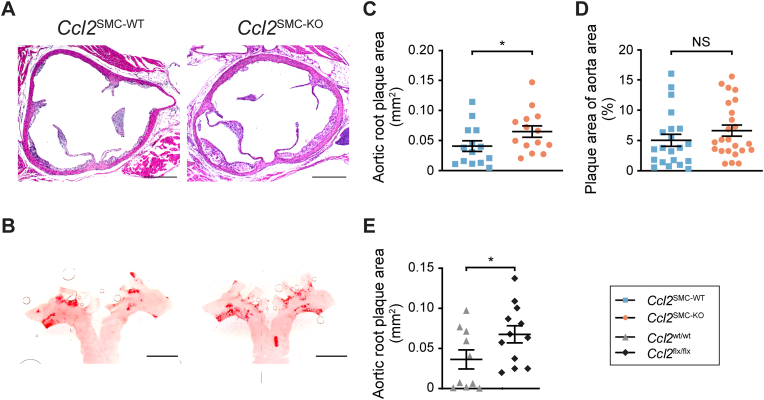
**Quantification of atherosclerosis after 12 weeks of atherogenic diet feeding**. Representative images of (A) hematoxylin-eosin-stained cross sections from the aortic root, and (B) Oil Red O-stained aortic arches opened for en face quantification. Scale bars = 400 μm (A) and 2 mm (B). (C) Plaque size was statistically significantly increased in *Ccl2*^SMC−KO^ (n = 14, orange circles) mice compared with *Ccl2*^SMC−WT^ (n = 14, blue squares) mice in the aortic root. (D) In the aortic arch, a similar tendency was seen but not statistically significant (*Ccl2*^SMC−KO^, n = 24; *Ccl2*^SMC−WT^, n = 21). Aortic root plaque size was also statistically significantly increased in *Cre*-negative *Ccl2*^flx/flx^ (black squares, n = 12) mice compared with *Ccl2*^wt/wt^ (grey triangles, n = 10) (E). Graphs show mean ± SEM. *p < 0.05, NS, not statistically significant by Welch *t*-test on log-transformed data. (For interpretation of the references to color in this figure legend, the reader is referred to the Web version of this article.)

Atherosclerosis development was assessed in sections of the aortic root and by en face analysis of the aortic arch after 12 weeks of hypercholesterolemia (Fig. 4A and B). Aortic root plaque area was significantly increased in *Ccl2*^SMC−KO^ compared with *Ccl2*^SMC−WT^ mice (Fig. 4C). In the aortic arch, the lesion coverage showed an insignificant tendency towards an increase in *Ccl2*^SMC−KO^ compared with wildtype littermates (Fig. 4D).

**Fig. 5:**
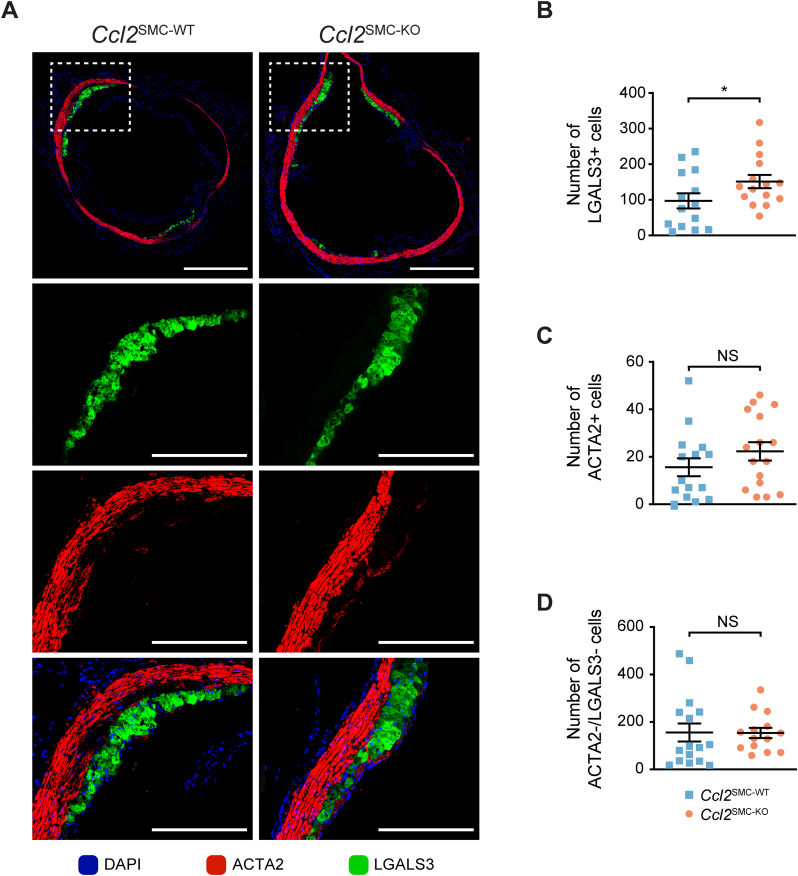
**Plaque composition in the aortic root after 12 weeks of atherosclerosis development.** (A) Representative images showing the entire aortic roots from *Ccl2*^SMC−WT^ and *Ccl2*^SMC−KO^ mice (top panel), and higher magnification views of the marked regions (dotted line boxes). LGALS3+ cells (green), ACTA2+ cells (red), and merged channels with DAPI (blue). Scale bars 200 μm for high magnification panels and 500 μm for images of the full aortic roots. Number of plaque LGALS3+ cells (*Ccl2*^SMC−WT^, n = 14; *Ccl2*^SMC−KO^, n = 15) (B), number of ACTA2+ cells (*Ccl2*^SMC−WT^, n = 15; *Ccl2*^SMC−KO^, n = 16) (C) and number of ACTA2/LGALS3 double-negative cells (*Ccl2*^SMC−WT^, n = 16; *Ccl2*^SMC−KO^, n = 14) (D) in aortic root sections from *Ccl2*^SMC−WT^ (blue squares) and *Ccl2*^SMC−KO^ (orange circles). Graphs show mean ± SEM. *p < 0.05; NS, not statistically significant by Welch *t*-test on log-transformed data. (For interpretation of the references to color in this figure legend, the reader is referred to the Web version of this article.)

Aortic root cross-sections were analyzed for plaque cellular composition using the macrophage marker LGALS3 and the SMC-marker ACTA2 (Fig. 5A). LGALS3 can be expressed on SMC lineage cells in addition to macrophages. Still, we have previously found that the level of misclassification by standard immune fluorescent staining in PCSK9-induced atherosclerosis is minor as assessed by SMC lineage tracing [26]. The number of LGALS3+ cells was increased in *Ccl2*^SMC−KO^ compared with wildtype littermates (Fig. 5B), while the number of ACTA2+ cells was unaffected (Fig. 5C).

Murine atherosclerotic lesions also contain a large population of SMC lineage cells that lose expression of ACTA2 and modulate to other forms [27]. We therefore also quantified ACTA2/LGALS3 double-negative cells (ACTA2-/LGALS3-), which are enriched for modulated plaque SMCs, but found no differences between the two groups (Fig. 5D).

None of the differences in atherosclerosis measures between groups were statistically significant in a multiple regression model incorporating final plasma cholesterol (data not shown) or the area-under-the-curve of plasma cholesterol levels from 2 until 11 weeks (Supplemental Tables 2 and 3). This indicates that the observed changes were explained by the unexpected differences in plasma cholesterol between groups, which was unrelated to the deletion of Ccl2 in SMCs. This was confirmed by analysis of aortic root plaque area in CreER^T2^-negative *Ccl2*^flx/flx^ and *Ccl2*^wt/wt^ mice (Fig. 4E). As observed for the *Ccl2*^SMC−KO^ mice, the plaque area was significantly increased in *Ccl2*^flx/flx^ mice compared to their wildtype counterparts, supporting the conclusion that secretion of CCL2 by modulated SMCs in the plaque is not an important mediator in plaque development.

## 4 Discussion

SMCs have traditionally been considered to have a protective role in atherosclerosis by producing fibrous tissue that stabilizes the plaque and protects it from rupture and thrombotic complications [28]. Over the last decade, however, multiple studies have revisited the role of SMCs in atherosclerosis using mouse models and lineage-tracing techniques [27]. Such experiments have shown that plaque SMCs derive from a small group of arterial SMCs that undergo clonal expansion and modulate to cells with fibroblast-like (aka. fibromyocytes) and chondrocyte-like (aka. chondromyocytes) phenotypes [[29], [30], [31], [32], [33]]. Some of these show evidence of inflammatory signaling [34], but the causal role of this for plaque development is still sparsely understood.

In the present study, we tested whether the ability of SMCs to secrete the proinflammatory cytokine CCL2 is a mediator linking SMC modulation to arterial inflammation and early plaque development. CCL2 is a central cytokine involved in monocyte recruitment to tissues, and global knockout of *Ccl2* reduces plaque formation and progression in mice [[5], [6], [7],^12^]. We confirmed the expression of CCL2 in human plaques in SMC-rich areas including the arterial media in the pre-diseased arterial wall. Furthermore, we confirmed the ability of both human and mouse SMCs to respond to an inflammatory stimulus with CCL2 expression. To test the potential causal role of this, we studied atherosclerosis in mice with SMC-specific conditional knockout of *Ccl2*.

Unexpectedly, we found increased hypercholesterolemia and atherosclerosis in mice with SMC-specific deficiency of *Ccl2* compared with wildtypes littermates. Importantly, however, a critical control experiment in mice lacking the Cre recombinase showed that this paradoxical effect was not caused by the knockout of *Ccl2* in SMCs. Rather it was explained by genetic differences between the floxed and wildtype *Ccl2* allele; either the floxing of the gene itself or passenger gene variants in linkage disequilibrium with the *Ccl2* locus originating from the 129/SVJ embryonic stem cells used to create the mouse line [18,35].

The genetic differences appeared to accelerate atherosclerosis at least partly by increasing hypercholesterolemia. Plasma cholesterol was significantly increased in *Ccl2*^SMC−KO^ compared with *Ccl2*^SMC−WT^ mice, which was unexpected since previous studies of mice with global CCL2 knockout or overexpression did not show any effects on plasma cholesterol levels [[5], [6], [7],^12^]. Although the differences in plasma cholesterol levels in *Ccl2*^flx/flx^ and *Ccl2*^wt/wt^ mice (without the *Cre* transgene) were not statistically significant, they resembled those of the main study. The differences were not caused by differences in the efficiency of rAAV-PCSK9 transduction since the effect was not present after 2 weeks but developed over time and plasma PCSK9 levels were similar between groups in the two genotypes.

Recently, Owsiany et al. reported increased atherosclerosis and macrophage recruitment in *Apoe*^−/−^ mice with heterozygous, but not homozygous, knockout of *Ccl2* in SMCs [16]. The increased atherosclerosis in heterozygous animals appeared to result from increased monocytosis in combination with a preserved ability to recruit monocytes into the arterial intima, which was lost in homozygous animals [16]. Recombination rates were not quantified in the study of Owsiany et al. and hence it is difficult to compare our mice (with an app. 75 % recombination rate of the floxed *Ccl2* locus) with the two genotypes reported in that study. Notably, even though our findings may on the surface seem consistent with the increased atherosclerosis reported in the heterozygous mice in Owsiany et al., the proatherogenic effect in our experiments, as discussed above, was not caused by *Ccl2* deletion. It is unlikely that the specific genetic background effects we saw in our study influenced the results of Owsiany et al., because they used a floxed *Ccl2-mCherry* mouse line, which was created using C57BL/6J embryonic stem cells [16,36]. In this line, mCherry is transcribed with *Ccl2* through a 2A aphtovirus cleavage site, and both transcripts are disrupted upon recombination. Pekayvaz et al. used the same *Ccl2-mCherry* line but found increased atherosclerotic plaque burden without monocytosis in mice with homozygous SMC-specific *Ccl2* deletion compared with littermate controls [14]. Reconciling the different findings from now three studies of SMC-specific *Ccl2* knockout is complicated. We think it reflects not only the complexity of the biology of SMC/pericyte-derived CCL2, which has different functions in the arterial wall, bone marrow, and potentially other organs, but also the complexity of the Cre-LoxP mouse technology and its pitfalls. To our knowledge, it has not been tested whether the *Ccl2-mCherry* allele (without recombination) affects atherosclerosis, as we found for the floxed *Ccl2* allele in the present study. This cannot be excluded since we have previously found that some SMC-expressed fluorescent proteins by themselves inhibit atherosclerosis [30].

Overall, we conclude that the knockout of *Ccl2* in SMCs in our experimental context did not change atherogenesis. This may indicate that SMC-secreted CCL2 is not an important regulator of plaque inflammation. Alternatively, it may be the result of multiple opposite effects of SMC-derived CCL2 on atherosclerosis that outbalance each other. It should also be noted that upregulation of both *Ccl7* (*Mcp3)* and *Ccl12* (*Mcp5)* located downstream of the *Ccl2* gene was originally demonstrated for the mouse line used in our studies upon Cre-mediated deletion of the floxed *Ccl2*-region [18]. Given that both encoded proteins are also ligands for the C–C chemokine receptor type 2 (CCR2), potential compensating effects from these genes cannot be excluded.

### 4.1 Strengths and limitations

Our study has limitations. Recombination of CCL2 alleles in SMCs was not complete as is generally the case in conditional knockout studies, and it is possible more robust inhibition of CCL2 secretion from SMCs would have affected plaque development. Despite multiple attempts with different antibodies, we did not succeed in performing immunostaining for CCL2 to evaluate whether the loss of SMC-derived CCL2 was consequential for total CCL2 content in plaques. Finally, our studies were not designed to investigate the impact of SMC-specific knockout on monocyte trafficking, which would have been relevant on the background of the recent opposing findings by Owsiany et al. [16] and Pekayvaz et al. [14].

## 5 Conclusion

Conditional deletion of *Ccl2* in SMCs does not inhibit the development of atherosclerosis in mice with PCSK9-induced hypercholesterolemia.

## Financial support

The study was funded by the Independent Research Fund Denmark (Sapere Aude II, 4004-00459), the 10.13039/100007405Danish Heart Foundation (17-R116-A7655-22072), and the 10.13039/501100009708Novo Nordisk Foundation (NNF17OC0030688). CNIC is supported by the Instituto de Salud Carlos III (ISCIII), the Ministerio de Ciencia e Innovación (MCIN) and the Pro CNIC Foundation, and is a Severo Ochoa Center of Excellence (grant CEX2020-001041-S funded by MICIN/AEI/10.13039/501100011033).

## Author contribution

Participated in research design: SG, JFB, Performed experiments and data analysis: SG, FL, UJFT, JTS, CBS, Writing or contributing to the writing of the manuscript: SG, UJFT, CBS, JFB, Review and final approval of the manuscript: SG, FL, UJFT, JTS, CBS, JFB.

## Declaration of competing interest

The authors declare that they have no known competing financial interests or personal relationships that could have appeared to influence the work reported in this paper.
